# Citronella essential oil-based nanoemulsion as a post-emergence natural herbicide

**DOI:** 10.1038/s41598-023-48328-6

**Published:** 2023-11-27

**Authors:** Naphat Somala, Chamroon Laosinwattana, Nawasit Chotsaeng, Montinee Teerarak

**Affiliations:** 1 School of Agricultural Technology, King Mongkut’s Institute of Technology Ladkrabang, Bangkok, 10520 Thailand; 2 Department of Chemistry, School of Science, King Mongkut’s Institute of Technology Ladkrabang, Bangkok, 10520 Thailand; 3 Advanced Pure and Applied Chemistry Research Unit (APAC), School of Science, King Mongkut’s Institute of Technology Ladkrabang, Bangkok, 10520 Thailand

**Keywords:** Chemical ecology, Mechanism of action

## Abstract

A natural herbicide nanoemulsion was fabricated from citronella (*Cymbopogon nardus* L.) essential oil (CEO) and a nonionic surfactant Tween 60 mixed with Span 60 at hydrophilic-lipophilic balance 14 using a microfluidization method. The main constituents of CEO were citronellol (35.244%), geraniol (21.906%), and citronellal (13.632%). CEO nanoemulsion droplet size and polydispersity index (PI) were evaluated by dynamic light scattering (DLS). The smallest droplet size (33.2 nm, PI 0.135) was obtained from a microfluidizer at 20,000 psi, 7 cycles. Nanoemulsion droplet in transmission electron microscopy correlated with DLS confirmed CEO to successfully produce nanoemulsion. The herbicidal activity of the nanoemulsion as a foliar spray was evaluated against *Echinochloa cruss-galli* and *Amaranthus tricolor* as representative narrow- and broadleaf weed plants, both of which presented visual toxicity symptoms. The modes of action of the nanoemulsion were then determined in terms of membrane integrity (relative electrolyte leakage; REL), malondialdehyde (MDA), and photosynthetic pigment contents. The results showed increase in REL and MDA which indicated the destruction of the treated plants; additionally, chlorophylls and carotenoid contents were decreased. Consequently, CEO nanoemulsion may have the possibility to act as a natural herbicide resource, and natural herbicides from citronella nanoemulsions could be good alternatives for use in sustainable agriculture.

## Introduction

Weeds are perennial problems in agricultural fields, and their management at present mainly involves using chemical herbicides for weed control^1^. Synthetic herbicides are the most cost-effective method for managing weeds, their widespread use damage consumer and farmer health^2^. Moreover, weeds are evolving resistant to chemical herbicides due to those herbicides having only a few modes of action (MOAs)^3^. Furthermore, some synthetic herbicides have been banned; for example, a myriad of countries have banned paraquat, including South Korea, European Union, and Austria. In 2016, China also restricted paraquat in an aqueous formulation^4^, and the Thai Ministry of Industry announced a complete ban on paraquat herbicides in June 2020. To achieve weed control alongside sustainable product and environmental safety, it is crucial to search for substitutes that can replace synthetic herbicides. The development of natural herbicides using secondary metabolites from plants is promoted as a sustainable alternative that can preserve the environment and farmer health^5^.

Essential oils (EOs) from plants are of widespread interest as potential bioherbicides^6–9^. These complex mixtures of volatile compounds act on multiple target molecules and so exert several MOAs in the recipient organism^6^. The herbicidal effect of EOs is reported to result in growth decrease, chlorosis, or leaf burning due to oxidative effect, electrolyte leakage, waxy cuticular coating reduction, cellular respiration decrease, photosynthetic pigment content decrease, or mitosis inhibition^10–12^. Due to their biocompatible, volatile, and environmental safety, EOs are more secure choices for farmers^13–15^; moreover, their diverse MOAs make it additionally difficult for weeds to evolve resistance^2^. The benefit of EO-based herbicides is a good prospect for sustainable agriculture.

Citronella (*Cymbopogon nardus*) is a perennial grass cultivated in Southeast Asia^16^. Ootani et al.^17^ reported herbicidal activities of Citronella EO (CEO) on *Digitaria horizontalis* and *Cenchrus echinatus* and showed that CEO induced severe injuries by disrupting membrane function, leading to increased membrane permeability and interrupting physiological and biological processes. The authors concluded that as CEO showed strong phytotoxic consequences on plant growth and reduced chlorophyll and protein contents, it could be promoted as a bioherbicide with various MOAs. CEO is registered in US EPA (The U.S. Environmental protection agency) as insect repellent due to its high efficacy, low toxicity and customer satisfaction (https://www3.epa.gov/pesticides/chem_search/reg_actions/reregistra- tion/red_PC-021901_30-May-97.pdf).

EO-based natural herbicides are usually formulated as oil-in-water (O/W) emulsions for convenience applications because EOs have weak water solubility and high volatility^5^. Increasingly, emulsions are formulated into nanoemulsions with small particle size (20–200 nm) to enhance their stability and efficacy^2^. The small droplet size allows the emulsion to be stable over a long timescale^18^. Methods of producing nanoemulsions can be mainly classified into two types, low- and high-energy emulsification methods^19^. Low-energy emulsification methods employ only chemicals and an ordinary stirring for the nanoemulsion fabrication^20^. These methods include phase inversion spontaneous emulsification, and solvent displacement^21^. However, low-energy methods require a high concentration of emulsification agents which may affect environmental safety. High energy methods are broadly utilized to fabricate nanoemulsion^20^. High-energy methods, such as ultrasonication, microfluidization, and high-pressure homogenization, require that a machine induces intense forces to fabricate smaller emulsion formulations. Among these methods, microfluidization is superior. Because it can generate emulsions with reduced droplet size and uniform size distribution^22^. This method generates narrower and smaller nanoemulsion droplet size distributions of than high-pressure homogenization. In addition, microfluidizer deliver stable nanoemulsions at low surfactant concentrations^20^. Therefore, microfluidization method was used for preparation in this study.

EO-based nanoemulsions have distinct benefits in terms of price and protection^23,24^. Because an O/W nanoemulsion is water-based, it requires considerably less organic solvent to produce than do conventional emulsifiable concentrates^23,25^. Additionally, the nanoscale size of droplets allows their uniform deposition on plant leaves, and the low surface tension of the system leads to increase wetting and permeation. The bioactive of water-insoluble pesticides is improved by solubilization in extremely small size of oil droplets; hence, nanoemulsion delivery systems are likely to improve pesticide efficacy^23^. In addition, Kaur, et al.^26^ suggested nanoemulsions as an achievable and efficient strategy for increasing the characteristic stability of bioactive ingredients, decreasing volatility, and preventing effects on the environment.

In a previous work, Somala, et al.^27^ formulated a CEO nanoemulsion using Tween 60 and Span 60 at a hydrophile-lipophile balance (HLB) 14 using a microfluidizer. This nanoemulsion had a pre-emergence herbicidal effect on *Echinochloa cruss-galli* seeds, completely inhibiting seed germination and seedling growth at 800 µL/L CEO. The treated seeds also showed decreased seed imbibition and α-amylase activity. To our best knowledge, this study builds on that work by investigating the CEO nanoemulsion as a post-emergence herbicide on *E. cruss-galli* and *Amaranthus tricolor*, and by studying the physiological mechanisms of its effect in terms of membrane integrity, malondialdehyde (MDA) content, and photosynthetic pigment content.

## Materials and methods

### Essential oil, chemical materials, and the tested weeds

Citronella EO was obtained from Thai–China Flavours and Fragrances Industry Co., Ltd. (Bangkok, Thailand). Tween 60 and Span 60 surfactants were obtained from Chemipan Corporation Co., Ltd. (Bangkok, Thailand). *Echinochloa crus-galli* (L.) Beauv. and *Amaranthus tricolor* were selected to represent narrowleaf and broadleaf weeds respectively. *Echinochloa crus-galli* was collected from natural fields and surrounding plateaus in the areas of King Mongkut’s Institute of Technology Ladkrabang University (KMITL), Thailand and cultured under the laboratory of the Department of Plant Production Technology, Faculty of Agricultural Technology of KMITL. *Amaranthus* tricolor, on the other hand, was obtained from Chia Tai Co., Ltd. and cultivated in a farmer's private farm in Thailand. The earlier samples were compared to type specimen *E crus-galli* (holotype K000245284) at Kew Herbarium (https://www.kew.org/). The latter materials were compared to type specimen *A. tricolor* (image of lectotype available at https://linnean-online.org/11633/). Both plant species were developed by Dr. Tiwtawat Napiroon, a botanist at the Faculty of Science and Technology, Thammasat University, Thailand and confirmed at the species level. Voucher specimens (collector no. SN-EC-001; SN-AT-001) of both plant types were deposited at the Department of Botany, Kasertsart University, Bangkok Forest Herbarium (BKF), Thailand. Herbarium acronyms follow Index Herbariorum (https://sweetgum.nybg.org/science/ih/). However, we confirm that these plants grow on agricultural area that is not covered by the Plant Variety Protection Act of Thailand or the International Union for Conservation of Nature (IUCN). The research on this plant species has comply with relevant institutional, national, and international guidelines and legislation.

### Gas chromatography/mass spectrometry (GC/MS) analyses of CEO constituents

The components of CEO were identified by GC–MS analyses. An Agilent series 6890N gas chromatography was coupled to an Agilent 5973 mass detector. The analysis was carried out on HP-5 silica capillary column (30 m; 0.25 mm ID; film thickness 0.25 µm). Operating conditions were as follows: oven temperature 40 C (3 min); 10 °C to 100 °C (5 min); 5–260 °C (5 min); flow rate of 1 ml/min of Helium gas. The sample volume (0.2 ml) was injected into the capillary column in the split mode (1:50). The temperature of ion source and interface was 230 °C and 280 °C, respectively. The identification of the compounds from CEO was performed according to their retention indices (RI), calculated by injecting a series of linear hydrocarbon standards of C8–C20 n-alkanes (Sigma-Aldrich, St. Louis, Missouri, USA) under the same conditions reported for CEO analysis. Individual constituents were distinguished via comparison of their mass spectra (molecular mass and fragmentation pattern) with those of the internal reference mass spectra library (National Institute of Standards and Technology, NIST, 2014) and approved by comparing their retention indices with those disclosed in the publications^28,29^. The relative amount of individual components of the total oil was expressed as a percentage peak area relative to total peak area.

### Preparation of the citronella EO-based nanoemulsion

CEO was prepared as an O/W nanoemulsion according to Somala et al.^27^ using a microfluidizer. The nanoemulsion consisted of 4% w/v CEO, 4% w/v surfactant mixture (Smix), and 96% w/v deionized (DI) water. The Smix consisted of 91.2% w/w Tween 60 (HLB 14.9) and 8.8% w/w Span 60 (HLB 4.7). All nanoemulsion formulations were kept at 4 °C until use.

### Nanoemulsion droplet size analysis

The dynamic light scattering (DLS) using a Nanoplus 3 (MICROMERITICS, Japan) was used for determination the droplet size, polydispersity index (PI) and zeta potential value of the CEO-based nanoemulsion. To avoid multiple scattering effects, the CEO-based nanoemulsion was diluted to 1:9 with deionized (DI) water before evaluation. The droplet size of emulsion was determined and computed using the program nanoPlus.

The transmission electron microscopy (TEM) (HITACHI HT7700, Japan) working at a voltage of 80 kV was used for determined morphology of nanoemulsion. In a negative staining procedure, a CEO nanoemulsion sample was diluted with DI water (1:9 v/v) and one droplet was added on a carbon-coated TEM grid for 10 min, fixed by 2% uranyl acetate and left for 30 s. The TEM grid was *allowed* to *dry* before TEM determination.

### Herbicidal activities of nanoemulsion

#### Preparation of the tested plants

Post-emergence herbicidal activities were evaluated by foliar spray under greenhouse conditions at an average temperature of 28–30 °C and relative humidity of 64–69%. Soil was prepared from soil, sand, and manure (3:1:1), and was used to fill plastic pots. Ten seeds of *E. crus-galli* and *A. tricolor* were sown in each pot at a depth of 1 cm from the soil surface and kept in an experimental house under natural light conditions. The planted pots were watered every day with tap water. Seedlings were thinned to four equal-sized plants per pot at seven days after sowing (DAS). The O/W nanoemulsion treatments applied to *E. crus-galli* incorporated 5, 10, 20, and 40 mL/L of EO, while those applied to *A. tricolor* contained 2.5, 5, 10, and 20 mL/L of EO. Water served as a control. All treatment solutions (including control) were sprayed at 21 DAS for *E. crus-galli* and 28 DAS for *A. tricolor* at a rate of 100 mL/m^2^ using a garden sprayer (model: SPRING SP01518-GE, Home Product Center Public Company Limited. Thailand).

#### Herbicidal activity

Visual toxicity symptoms were evaluated in *E. crus-galli* and *A. tricolor* at 1, 7, 14, and 21 days after treatment. Plants were scored on a 1–10 scale where ‘1’ means without toxicity symptoms and ‘10’ means 100% complete plant death^5^. Twenty-one days after treatment, relative electrolyte leakage (REL), malondialdehyde (MDA) content, and photosynthetic pigment contents were assessed.

#### Relative electrolyte leakage (REL)

REL was evaluated in fresh leaves of the treated weeds by the method of Singh et al.^30^ with modification. Electrolyte leakage conductivity was measured when five fresh leaf discs were floated on 10 mL of water after 1 h at room temperature (EC1) and after boiling at 100 °C for 20 min (EC2). Measurement was conducted using a Consort C3010 multi-parameter analyzer (Consort, Belgium). REL was calculated by the formula:

$$ \% {\text{ REL}} = \left( {{\text{EC1}}/{\text{EC2}}} \right) \times {1}00 $$

#### Malondialdehyde (MDA) content

MDA is a free radical and a final product of the lipid peroxidation process^31^. Its abundance was indicated by the concentration of thiobarbituric acid reactive substances (TBARs). A sample of treated leaves (0.5 g) was ground in 0.1% (w/v) trichloroacetic acid, then centrifuged (6000×*g*) for 20 min and the supernatant was collected. A reaction solution was then prepared, consisting of supernatant, 0.5%, w/v thiobarbaturic acid and 4% w/v butylhydroxytoluene. The reaction mixture solution was boiled at 95 °C for 30 min and then centrifuged at 6000×*g* at 4 °C for 10 min. The absorbance was recorded at 532 and 600 nm, and the TBAR concentration was estimated using an extinction coefficient of 155 mM/cm. The results were described as nmol/g on the basis of fresh weight^32^.

#### Photosynthetic pigments

Fresh leaves (0.1 g) from treated plants were ground in aqueous 80% acetone and incubated in the dark box at room temperature for 3 h. Chlorophylls and carotenoid contents were evaluated by measuring absorbance with a UV/vis spectrophotometer at 663, 647, and 470 nm and calculating concentrations according to Lichtenthaler’s equation^33^.

### Statistical analysis

All experiments were repeated at least three times. The experiments were organized in a completely randomized design (CRD) with 4 replications. Results are presented as mean ± standard deviation. All data were subjected to analysis of variance (ANOVA) and a pairwise comparison of mean by Tukey’s test (*p* ≤ 0.05) using SAS version 9.00. Means followed by the same letter(s) are not significant.

## Results and discussion

### Chemical constituents of CEO

GC–MS analysis identified 21 components comprising 97.282% of the total CEO. The major constituents (83.254%) belonged to the monoterpene class and consisted mainly of citronellol (35.244%), geraniol (21.906%), and citronellal (13.632%) (Table 1). EOs are established to be complex compound combinations containing about 20–60 components^34^; furthermore, our findings are in agreement with Timung et al.^35^, who identified citronellal, geraniol, and citronellol as the main compounds of CEO. On the other hand, Nakahara et al.^16^ presented primary constituents of geranyl acetate, *trans*-citral, geraniol, *cis*-citral, citronellal, and citronellol. However, numerous aspects such as climatic, seasonal, genetic variations, and harvest stage can affect the chemical constituents of EOs^36^. Also, Kaur et al.^14^ reported that citronella EO chemical composition varies with environmental factors, climatic conditions, and harvest time. In addition, Silva Lima et al.^37^ reported that the effect of seasonal factors on the chemical composition of the *Ocimum gratissimum* EO can interfere acaricidal activity of *O. gratissimum* EO. This EO from plants that were harvested in the rainy season presented lower acaricidal activity. Therefore, the application of plant EO-based nanoemulsion should be carefully used because various factors influence the chemical composition of EO.

**Table 1:** Constituent of essential oil from citronella leaves.

Number	Class	Constituent	Formula	RT^a^ min	RI^b^	% of total CEO^c^
1	Monoterpene	Limonene	C_10_H_16_	6.374	1030	3.863
2		Linalool	C_10_H_18_O	6.949	1100	0.897
3		Citronellal	C_10_H_18_O	7.394	1142	35.244
4		Isopulegol	C_10_H_18_O	7.494	1147	0.378
5		Decanal	C_10_H_20_O	7.771	1186	0.116
6		Citronellol	C_10_H_20_O	7.947	1213	13.632
7		Neral	C_10_H_16_O	8.083	1223	0.145
8		Geraniol	C_10_H_18_O	8.152	1238	21.906
9		Geranial	C_10_H_16_O	8.287	1239	0.306
10		*Eugenal*	C_10_H_12_O_2_	8.818	1342	2.999
11		Citronellylacetate	C_12_H_22_O_2_	8.932	1335	0.901
12		Geranylacetate	C_12_H_20_O_2_	9.025	1366	2.867
13	Sesquiterpene	*ß*-Elemene	C_15_H_24_	9.205	1390	3.120
14		*α*-Humulene	C_15_H_24_	9.674	1454	0.272
15		γ-Muurolene	C_15_H_24_	9.771	1478	0.495
16		Germacrene D	C_15_H_24_	9.837	1498	2.515
17		δ-Cadinene	C_15_H_24_	10.044	1515	3.227
18		Naphthalene, 1,2,3,5,6,8a-hexahydro-4,7-dimethyl-1- (1-methylethyl)-	C_15_H_24_	10.149	1492	0.102
19		Elemol	C_15_H_26_O	10.217	1540	3.314
20		Farnesol	C_15_H_26_O	10.54	1698	0.100
21	Oxygenated sesquiterpene	*τ*-Cadinol	C_15_H_26_O	10.793	1789	0.883
	Monoterpene					83.254
	Sesquiterpene					13.145
	Oxygenated sesquiterpene					0.883
	Total					97.282

^a^Retention time.

^b^Retention indices relative to C8-C20 n-alkanes on HP-5MS capillary column.

^c^Relative area percentage (peak area relative to the total peak area, %).

With regard to the herbicidal effect of CEO constituents, Choi, et al.^38^ reported a strong inhibitory effect of palmarosa oil, with the main component geraniol, on seed germination and seedling development of *Echinochloa crus-galli, Aeschynomene indica* L., and *Brassica napus* L. Examining the effects of individual essential oil components, they found geraniol to exert significantly higher inhibitory activity on *E. crus-galli*. Lins et al.^11^ reported that citronellol enhances solute leakage and induces reactive oxygen species (ROS) generation, which could result in lipid peroxidation and membrane damage. The authors also showed that citronellal can inhibit seed germination and seedling growth, affecting plant roots and shoots.

### Influence of microfluidization conditions on CEO nanoemulsion droplet size

**Figure 1:**
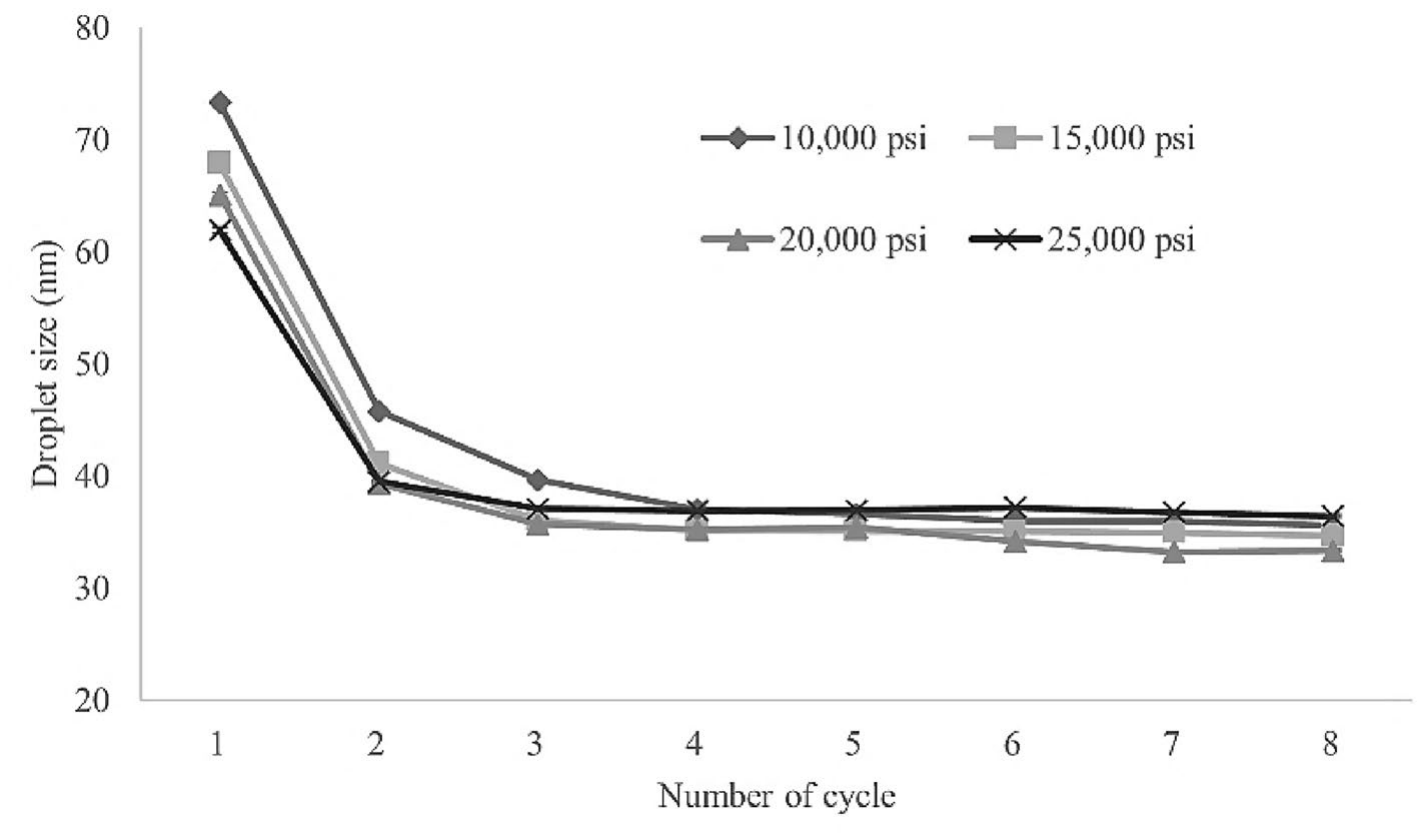
Influence of functional pressure and cycle number on droplet size of citronella essential oil nanoemulsion prepared with surfactant mixture (Tween 60 and Span 60) at a concentration of 4% (w/v).

The high intensity of the shear forces and turbulence produced during microfluidization, which are pressure dependent, can affect droplet size and size distribution and hence significantly impact the emulsion’s physical characteristics^22^. Here, the influence of pressure and cycle number on the CEO nanoemulsion was evaluated for pressures from 10,000 to 25,000 psi with 1–8 cycles. Coarse emulsion’s droplet size was 163.3 ± 2.0 nm; the results of the microfluidization are presented in Fig. 1. In short, increasing pressure enhances droplet deformation and subsequent disruption, leading to lower droplet size. In addition, droplet size decreased as cycle number increased. When the coarse emulsion was homogenized for one cycle at 10,000 psi, 15,000 psi, 20,000 psi, and 25,000 psi, the means droplet size decreased from 73.3 ± 0.5 to 67.9 ± 0.7 to 65.1 ± 0.5 and finally 61.9 ± 0.5 nm. The smallest droplet size (33.2 ± 0.3 nm) was produced when applying the microfluidizer at 20,000 psi for 7 cycles.

Table 2 presents the effect of microfluidization parameters on the nanoemulsion PI, which indicates homogeneity. At all pressures, PI values were markedly reduced when performing more than 2 cycles of homogenization. The PI value for the smallest droplet size of CEO nanoemulsion (7 cycles at 20,000 psi) was 0.135.

**Table 2:** Influence of operating pressure and cycle number on polydispersity index (PI) of citronella essential oil nanoemulsion prepared with surfactant mixture (Tween 60 and Span 60) at a concentration of 4% (w/v).

Number of cycles	Pressure (psi)			
	10,000	15,000	20,000	25,000
1	0.247 ± 0.006 a	0.265 ± 0.006 a	0.267 ± 0.007 a	0.263 ± 0.002 a
2	0.183 ± 0.008 b	0.178 ± 0.014 b	0.162 ± 0.012 b	0.154 ± 0.009 b
3	0.134 ± 0.016 c	0.142 ± 0.011 c	0.124 ± 0.014 cd	0.122 ± 0.009 cd
4	0.115 ± 0.009 cd	0.179 ± 0.029 b	0.117 ± 0.007 cd	0.104 ± 0.003 d
5	0.124 ± 0.013 cd	0.125 ± 0.015 c	0.106 ± 0.021 d	0.116 ± 0.015 cd
6	0.120 ± 0.012 cd	0.111 ± 0.016 c	0.126 ± 0.017 cd	0.135 ± 0.021 bc
7	0.108 ± 0.013 d	0.126 ± 0.019 c	0.135 ± 0.005 bcd	0.107 ± 0.007 d
8	0.105 ± 0.014 d	0.118 ± 0.010 c	0.137 ± 0.023 bc	0.130 ± 0.009 c

Means ± standard deviation. Means with different letters within a column are significantly different as indicated by Tukey’s test (*p* ≤ 0.05).

Our findings are in agreement with a prior research that tocotrienol rich fraction nanoemulsions obtained after 10 cycles of homogenization show reduced droplet size with increasing pressure^39^. In addition, Jintanasirinurak et al.^40^ fabricated CEO nanoemulsions using a microfluidizer at 15,000 psi for 1–3 cycles and obtained the smallest droplet size at 3 cycles. Here, the optimal microfluidization conditions (smallest droplets) were 20,000 psi for 7 cycles; accordingly, this formulation was used in further experiments.

### Nanoemulsion characteristics

The droplet size, PI, and zeta potential value of the optimized nanoemulsion formulation were determined by DLS (Table 3). The droplet size of 33.2 nm is within the nanoemulsion scale (20–200 nm)^2^. The PI value indicates a narrow size distribution with uniform droplets. The zeta potential value of >+ 30 or <− 30 mV confirms that representing a high energy barrier toward droplet coalescence and hence good stability of the nanoemulsion. These results confirm the formation of a nanoemulsion. One of the essential characteristics of nanoemulsions is the stability^41^. A previous study that used similar protocols to ours supports that surfactant Tween 60 and Span 60 ensure the stability of emulsions prepared from CEO. The stability of CEO nanoemulsion was investigated under various conditions (temperatures of 4, 25, and 45 °C for 0–28 days). The suitable condition of CEO nano-formulation was 4 °C without increased droplet size for 28 days^27^. Gharsan et al.^36^ also fabricated citronella nanoemulsions with a nonionic surfactant (Tween 80) and determined that droplet stability is because the surfactant reducing the free interface energy, which provides a mechanical barrier to the incorporation. In addition, the reduced droplet size could enhance EO solubility, which would also increase solution stability.

**Table 3:** Characteristics of the citronella essential oil nanoemulsion prepared using the microfluidizer at 20,000 psi for 7 cycles with surfactant mixture (Tween 60 and Span 60) at a concentration of 4% (w/v).

Droplet size (nm)	PI value	Zeta potential (mV)
33.2 ± 0.3	0.135 ± 0.005	− 35.42 ± 4.08

Means ± standard deviations.

**Figure 2:**
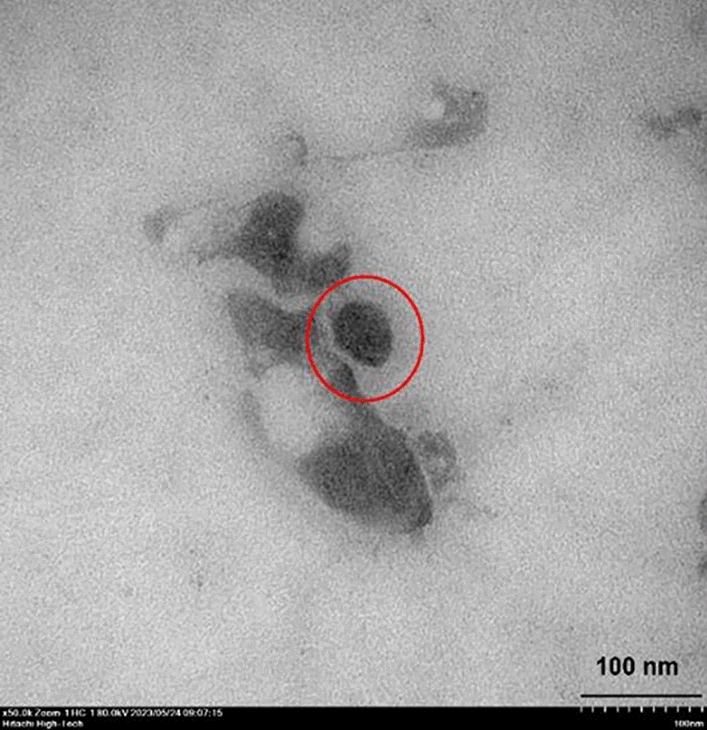
Transmission electron microscopy of the citronella essential oil nanoemulsion prepared using the microfluidizer at 20,000 psi for 7 cycles with surfactant mixture (Tween 60 and Span 60) at a concentration of 4% (w/v). The red circle shows the nanoemulsion droplet. Scale bar, 100 nm.

Droplet shape was visually examined by transmission electron microscopy (TEM) (Fig. 2). Droplets of the oil and surfactant mixture appeared as black circles with diameter < 100 nm, which correlated with the droplet size of the nanoemulsion as determined by DLS. Also, Sharma, et al.^42^ founded that TEM image of the nanoemulsion from clove oil was spherical nanoemulsion droplets.

### Herbicidal effect of CEO nanoemulsion

**Figure 3:**
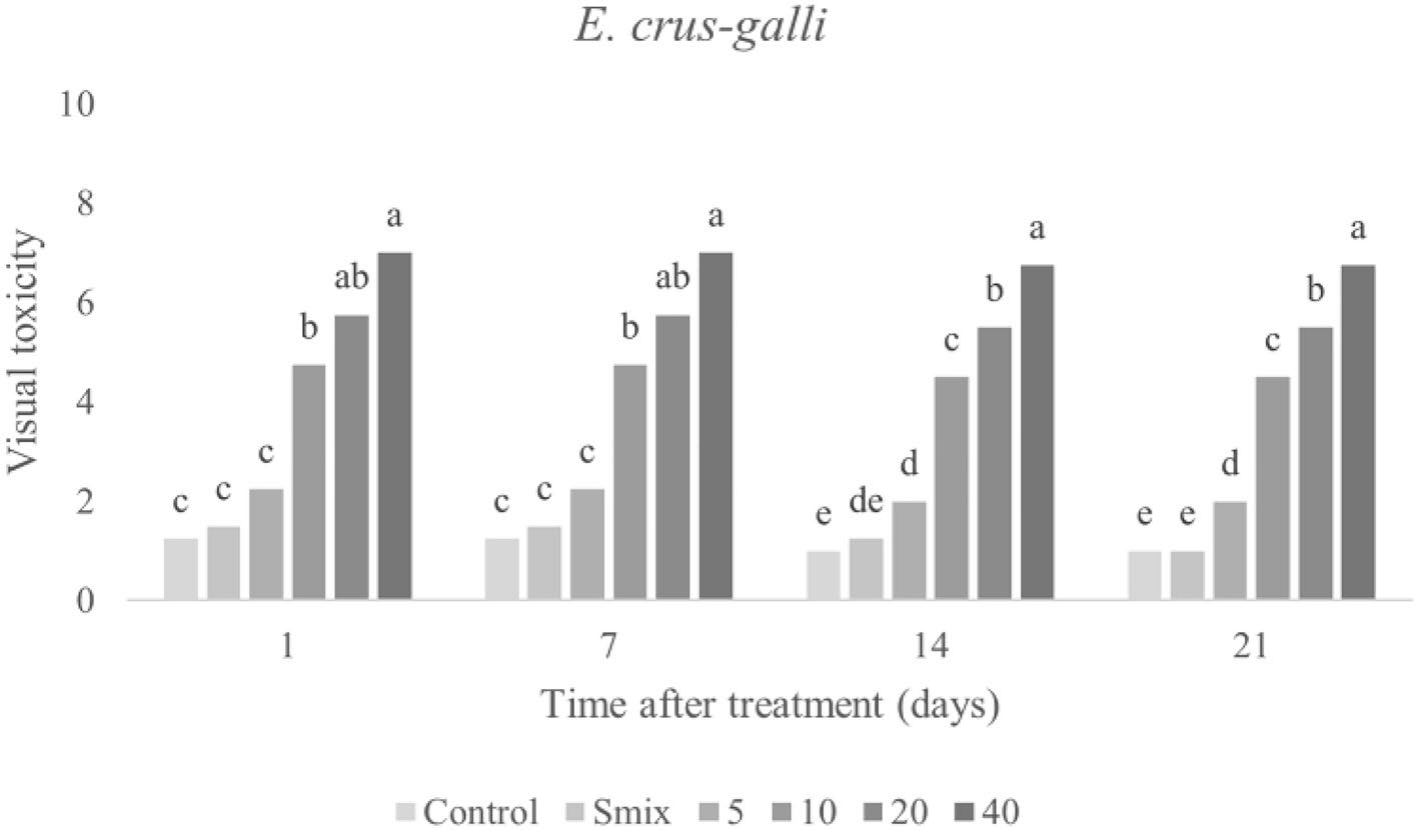
Visual toxicity scores of *E. crus-galli* leaves treated with surfactant mixture (Smix) alone (40 mL/L) and citronella essential oil nanoemulsion at different concentrations of citronella essential oil (5–40 mL/L) prepared using the microfluidizer at 20,000 psi for 7 cycles with surfactant mixture (Tween 60 and Span 60). Means with different letters within time after treatment are significantly different as indicated by Tukey’s test (*p* ≤ 0.05).

**Figure 4:**
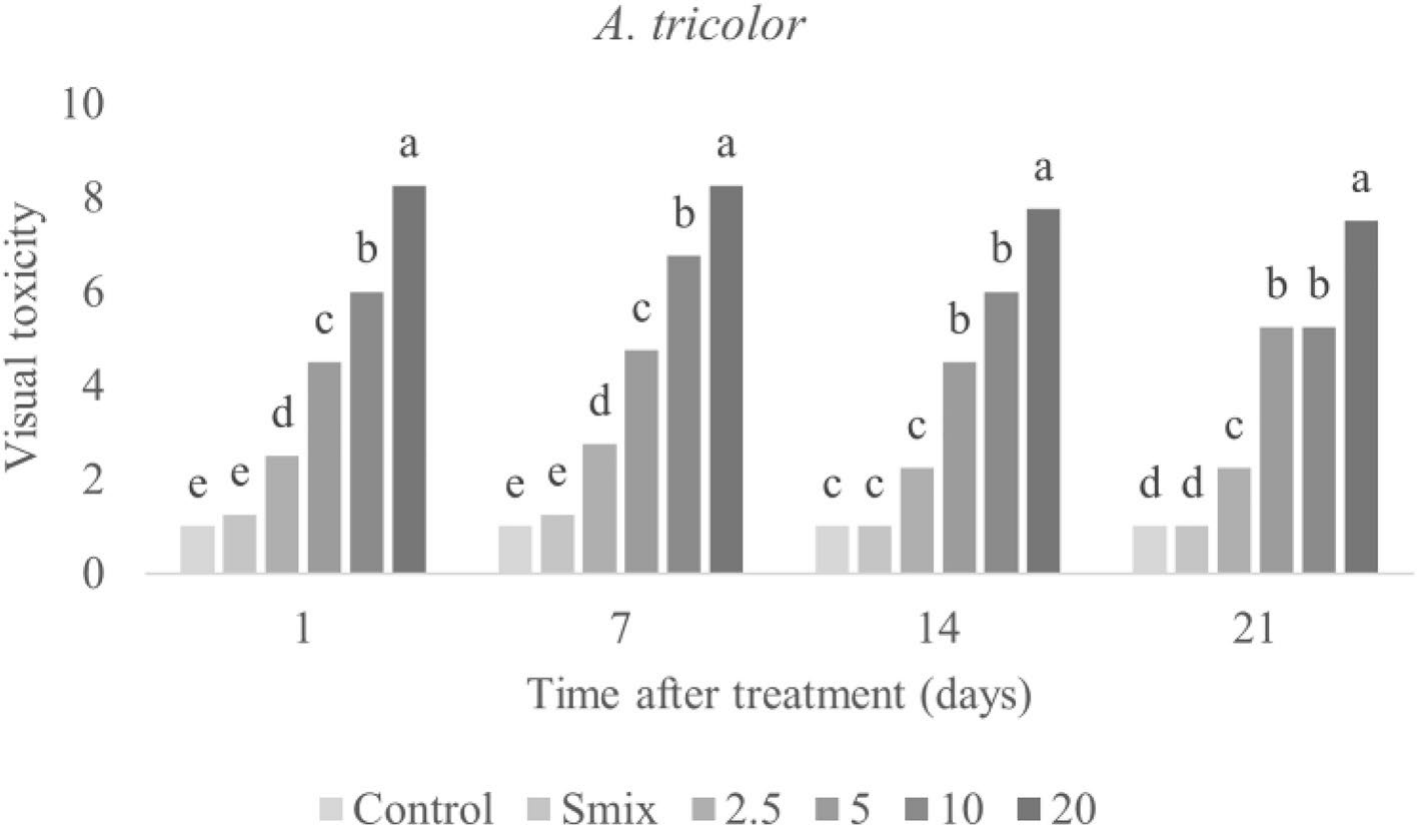
Visual toxicity scores of *A. tricolor* leaves treated with surfactant mixture (Smix) (20 mL/L) and citronella essential oil nanoemulsion at different concentrations of citronella essential oil (2.5–20 mL/L) prepared using the microfluidizer at 20,000 psi for 7 cycles with surfactant mixture (Tween 60 and Span 60). Means with different letters within time after treatment are significantly different as indicated by Tukey’s test (*p* ≤ 0.05).

**Figure 5:**
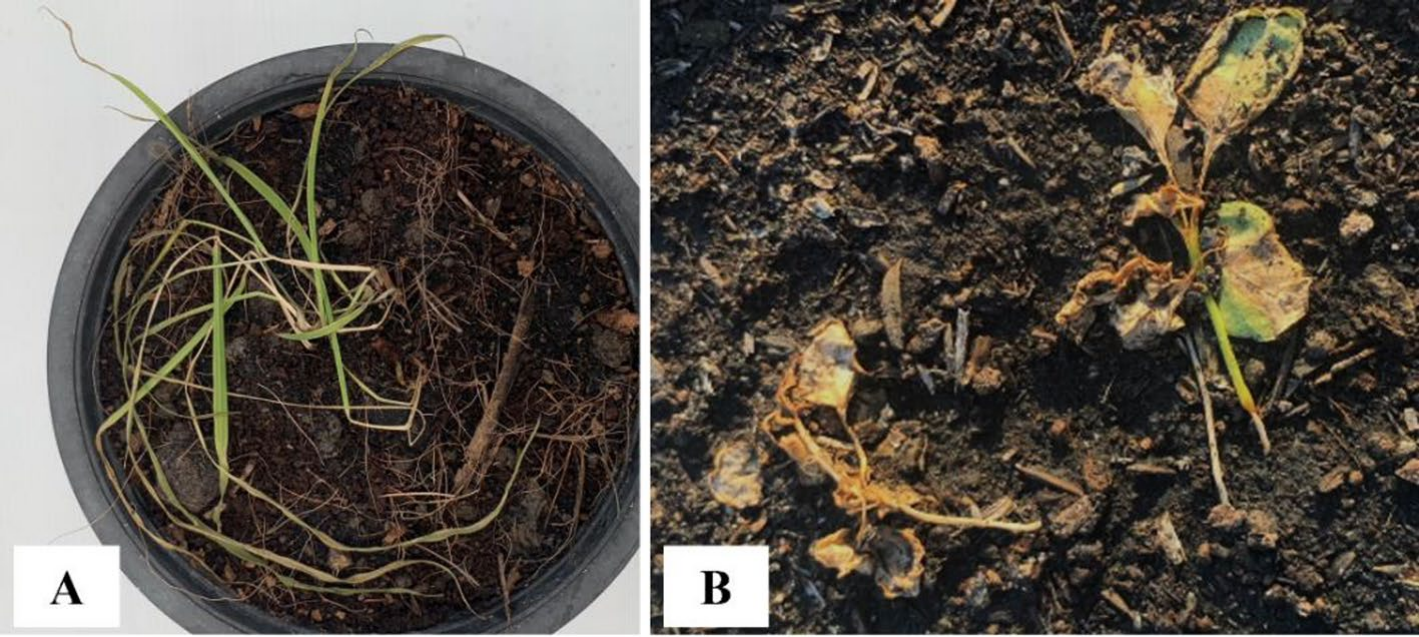
Representative images of toxicity injuries 21 days after treatment with citronella essential oil nanoemulsion at a highest concentration of *E. cruss-galli* (**A**) and *A. tricolor* (**B**) prepared using the microfluidizer at 20,000 psi for 7 cycles with surfactant mixture (Tween 60 and Span 60).

Visual toxicity symptoms were observed at 1, 7, 14, and 21 days after nanoemulsion application to *E. crus-galli* and *A. tricolor* leaves. Figures 3 and 4 plot the toxicity scores, and show increased symptoms with increasing concentrations of nanoemulsion. The highest visual toxicity score of the nanoemulsion was obtained from the highest concentration in both treated plants. In the case of *E. crus-galli*, visual toxicity of the nanoemulsion at a concentration of 20 mL/L of CEO was 5.75 out of 10 after 1 day after treatment. At the highest concentration (40 mL/L), visual toxicity of the nanoemulsion was 7 out of 10 after 1 day after treatment. However, the visual toxicity of the nanoemulsion in *A. tricolor* was 8.25 out of 10 at the highest concentration (20 mL/L). The visual toxicity scores: 0 = no effect (normal), 1–3 = slight effect (slight injury or discoloration, little stunting and some stand loss), 4–6 = moderate effect (moderate injury, recovery possible and near-severe harm), 7–9 = Severe effect (serious damage, stand loss, almost total destruction and a few remaining plants), 10 = complete (total plant death). As a result, *A. tricolor* may be sensitive to CEO nanoemulsion more than *E. crus-galli*. *E. crus-galli* symptoms were leaf burning, wilting, and necrosis (Fig. 5A), while *A. tricolor* showed leaf burning, leaf folding, wilting, and necrosis (Fig. 5B) in agreement with the literature that herbicidal effects of EOs on weed plants presented severe growth decrease, chlorosis, or leaf burning^11^. The previous study to use a CEO-based nanoemulsion focused on pre-emergence herbicidal activity^27^, and observed inhibited germination and seedling growth. Our observations of post-emergence toxicity symptoms support that CEO, with its complex mixture of volatile compounds, can have multiple mechanisms of action. Similarly, Poonpaiboonpipat, et al.^43^ studied the effect of lemongrass (*Cymbopogon citratus*) EO applied via foliar spray to *E. crus-galli* in concentrations of 1.25, 2.5, 5 and 10% (v/v), and indicated both pre- and post-emergence herbicidal activities. As their results, major compounds of *C. citratus* EO were monoterpene in agreement with our study that had herbicidal activities on *E. crus-galli*.

#### Relative electrolyte leakage (REL)

**Figure 6:**
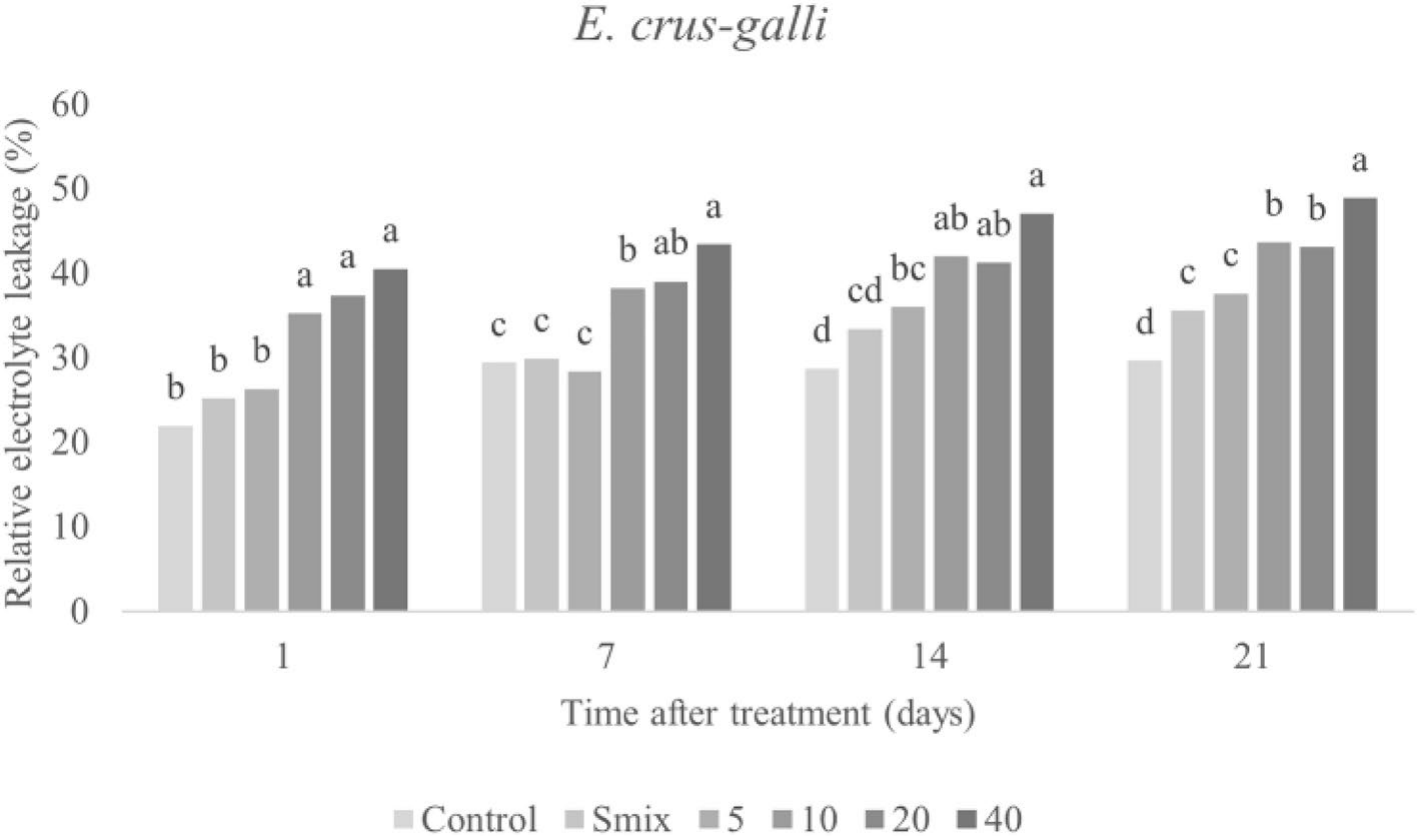
Relative electrolyte leakage of *E. crus-galli* leaves treated with surfactant mixture (Smix) (40 mL/L) and citronella essential oil nanoemulsion at different concentrations of citronella essential oil (5–40 mL/L) prepared using the microfluidizer at 20,000 psi for 7 cycles with surfactant mixture (Tween 60 and Span 60). Means with different letters within time after treatment are significantly different as indicated by Tukey’s test (*p* ≤ 0.05).

**Figure 7:**
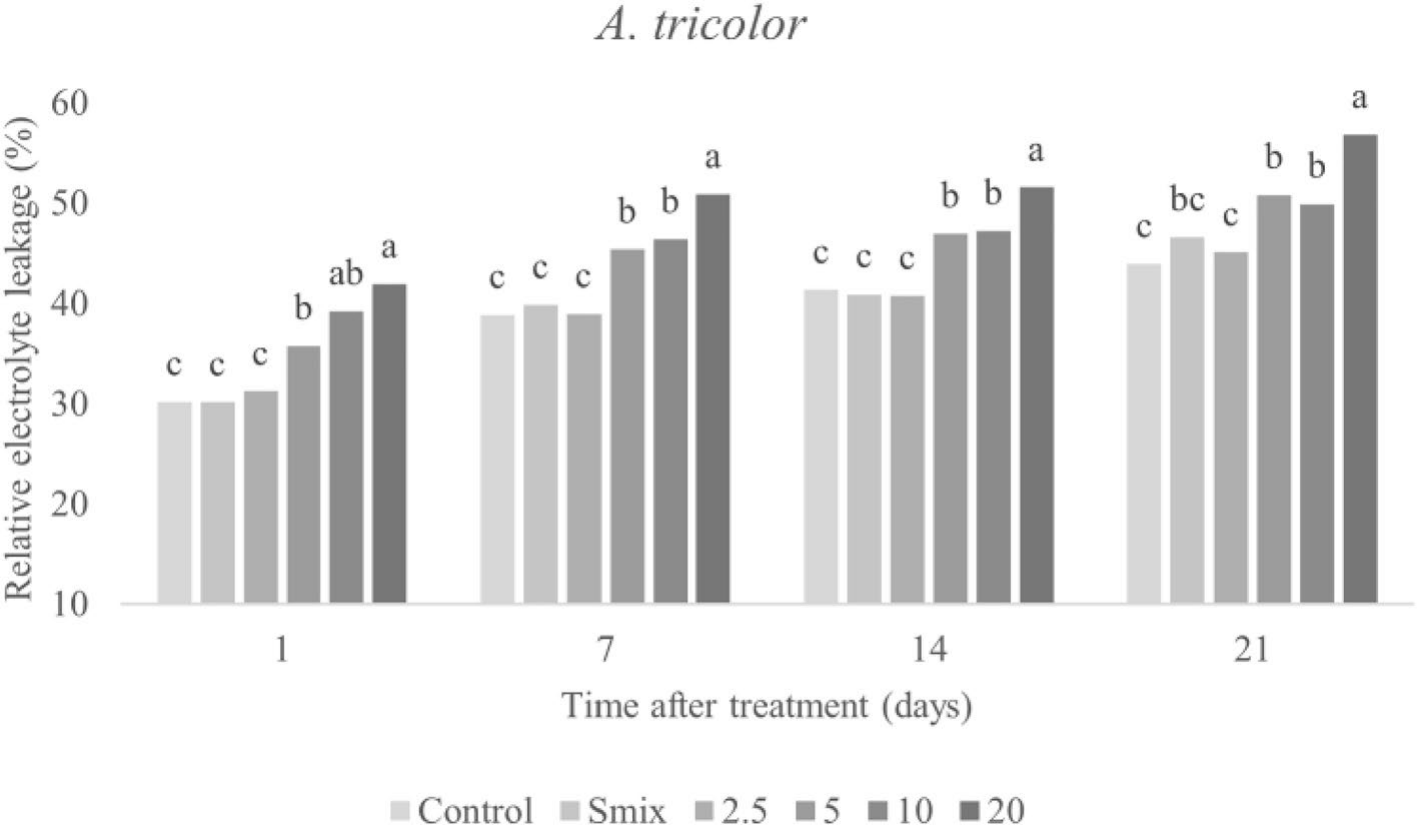
Relative electrolyte leakage of *A. tricolor* leaves treated with surfactant mixture (Smix) (20 mL/L) and citronella essential oil nanoemulsion at different concentrations of citronella essential oil (2.5–20 mL/L) prepared using the microfluidizer at 20,000 psi for 7 cycles with surfactant mixture (Tween 60 and Span 60). Means with different letters within time after treatment are significantly different as indicated by Tukey’s test (*p* ≤ 0.05).

As shown in Figs. 6 and 7, REL was significantly increased in *E. crus-galli* and *A. tricolor* leaves after exposure to the CEO nanoemulsion. In both species, the strongest effect was observed on day 21 with the highest treatment concentration (48.88% REL in E. crus-galli, 56.83% in A. tricolor). Increased REL percentage is indicative of membrane damage. CEO nanoemulsions are known to disrupt membrane function, which enhances permeability and, cell component and electrolyte leakage^2^. Leakage may also occur due to uncontrolled production and accumulation of reactive oxygen species (ROS)^44,45^. Another, Taban, et al.^5^ likewise produced a nano-encapsulation of savory (*Satureja hortensis*) EO and showed it to induce membrane leakage in *Amaranthus retroflexus* L. Pouresmaeil, et al.^45^ similarly determined that *Artemisia fragrans* EO caused accumulation of H_2_O_2_, which induced lipid peroxidation that affected the membrane bilayer phospholipids. Importantly, any change in permeability could affect biochemical and physiological processes connected to membrane operation^2^. Loss of membrane function affects cell components and metabolism processes, ultimately resulting in slow plant development and finally cell death^46^. Furthermore, nanoemulsion droplets reportedly penetrate into cells, resulting in the loss of the spaces between them, and disrupt cell connection. Additionally, disruption of cell membranes results in cell coagulation, in which cellular components are released into the outside of the cells and, finally, tissues are destroyed^2^.

Notably, the major constituents of CEO are predominantly monoterpenes. The biological activities of these mostly lipophilic compounds are mediated via lipid packing density and liquidity, direct linking with the lipid bilayer, and alteration of the physical structures of membrane components^2,47^. Additionally, the major component geraniol is a terpenoid phenol. Zhang, et al.^48^ reported a nanoemulsion of another terpenoid phenol, carvacrol, to damage the phospholipid bilayers of plant cell membranes. Taken together, these findings support that CEO-based nanoemulsions may disrupt plant cell membranes and destroy cell components.

#### Malondialdehyde (MDA) content

**Figure 8:**
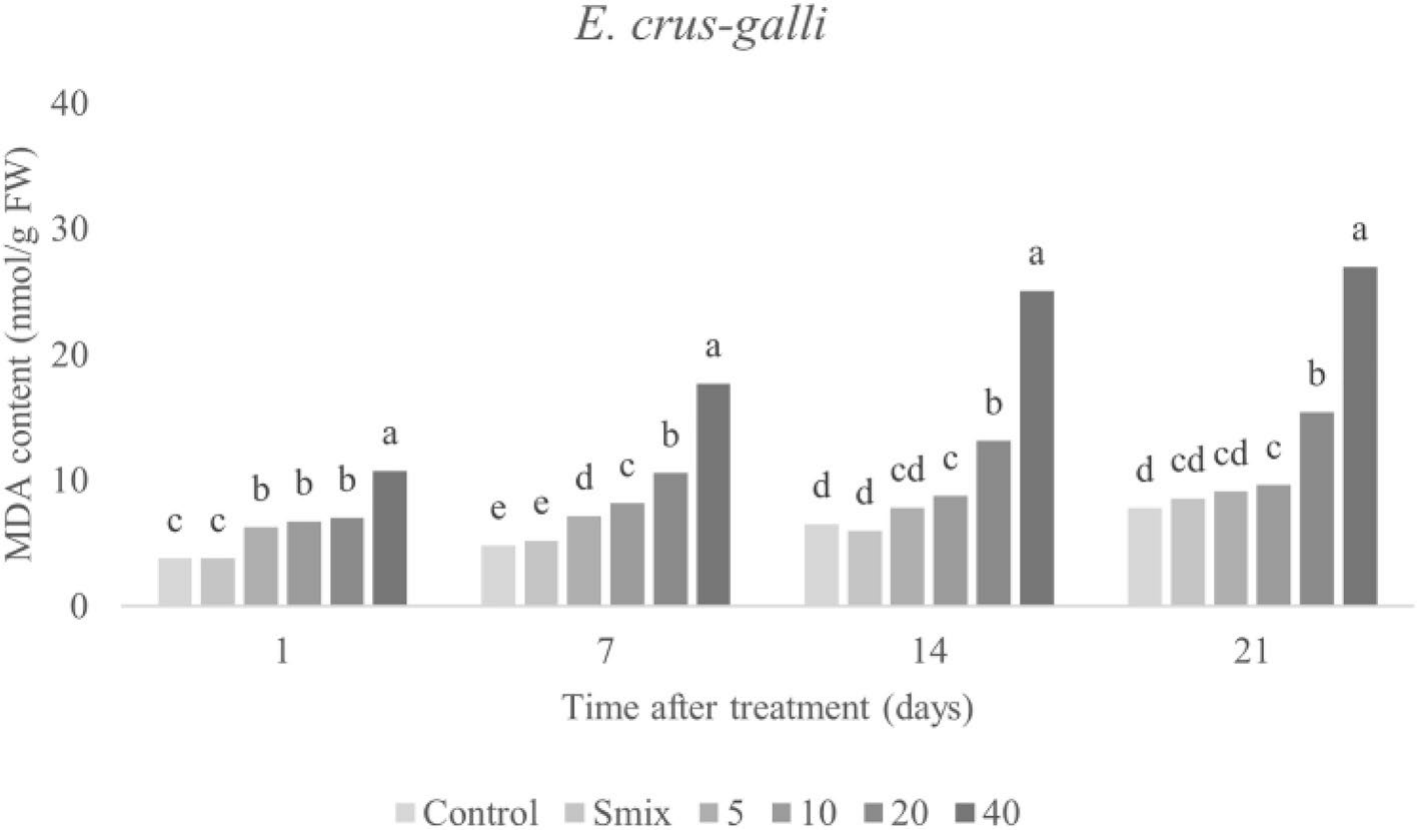
Malondialdehyde (MDA) content in *E. crus-galli* leaves treated with surfactant mixture (Smix) (40 mL/L) and citronella essential oil nanoemulsion at different concentrations of citronella essential oil (5–40 mL/L) prepared using the microfluidizer at 20,000 psi for 7 cycles with surfactant mixture (Tween 60 and Span 60). Means with different letters within time after treatment are significantly different as indicated by Tukey’s test (*p* ≤ 0.05).

**Figure 9:**
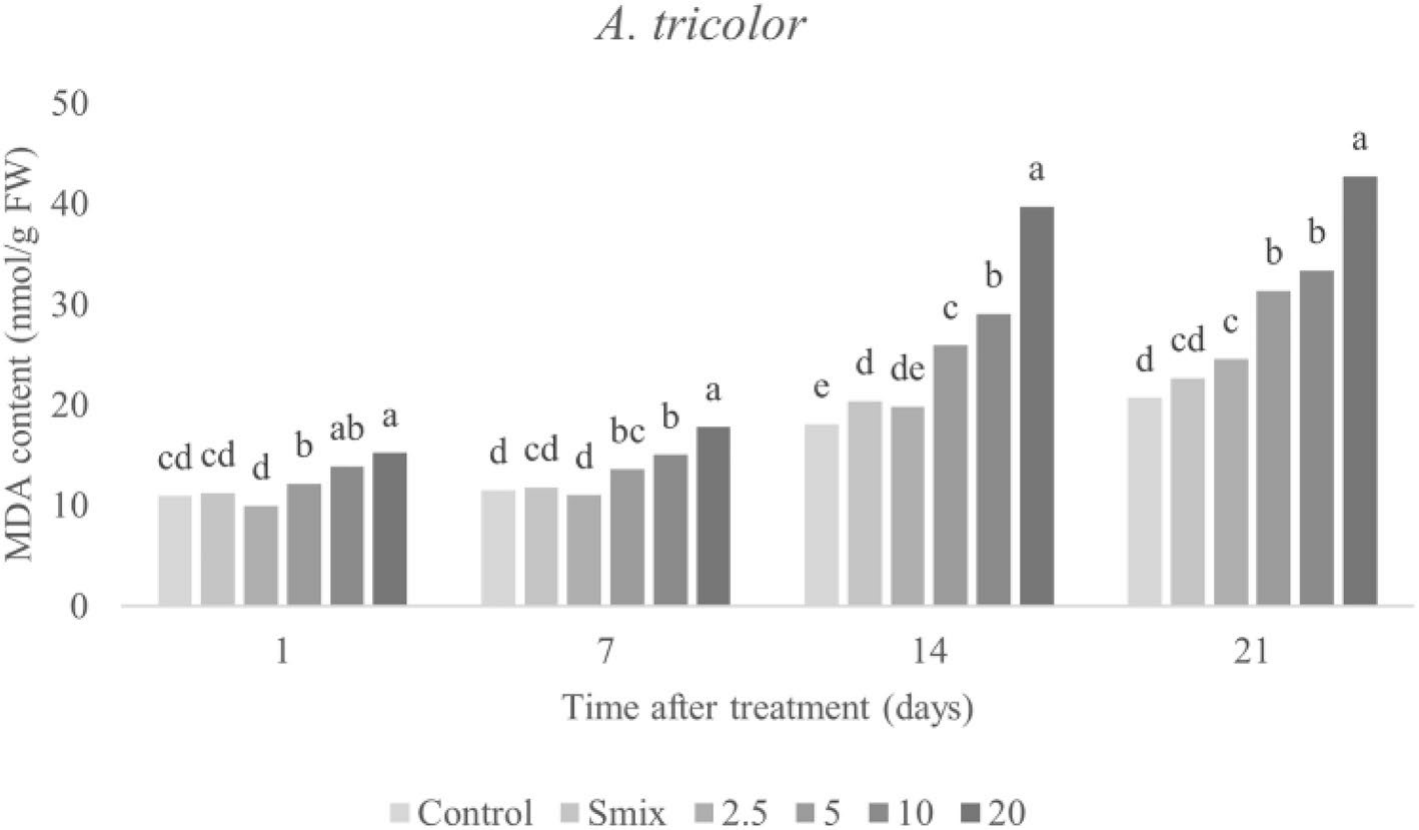
Malondialdehyde (MDA) content in *A. tricolor* leaves treated with surfactant mixture (Smix) (20 mL/L) and citronella essential oil nanoemulsion at different concentrations of citronella essential oil (2.5–20 mL/L) prepared using the microfluidizer at 20,000 psi for 7 cycles with surfactant mixture (Tween 60 and Span 60). Means with different letters within time after treatment are significantly different as indicated by Tukey’s test (*p* ≤ 0.05).

MDA is a secondary end product of lipid peroxidation of polyunsaturated fatty acids^49^, and is assayed to determine lipid peroxidation in cell membranes of plant. MDA content in the treated leaves was affected by CEO treatment in a concentration-dependent manner (Fig. 8), with the highest value of 10.74 nmol/g FW observed 21 days after treatment of the nanoemulsion with the highest tested concentration (40 mL/L). A similar trend was observed in *A. tricolor* (Fig. 9), with a peak of 42.73 nmol/g FW observed 21 days after treatment of the nanoemulsion with the highest concentration (20 mL/L). Accumulation of MDA content correlated with membrane leakage, detailed above. As fatty acids are components of cell membrane of plants, oxidative action against lipids can degrade membranes, producing free lipids^46^. As mentioned above, increasing ROS can result in greater lipid peroxidation, measurable as MDA content. The observed increase in MDA content alongside increasing membrane leakage indicates that the CEO-based nanoemulsion induced lipid peroxidation stress, ultimately leading to loss of membrane operation^50,51^. These consequences are highly consistent with previous work revealing that natural herbicides developed from essential oils have negative effects on lipids in weed plants^5,46,52^. Ootani et al.^17^ presented that EOs can eradicate membrane integrity and therefore enhance permeability, as well as promote oxidation of cellular structures, including membrane lipids. More, monoterpene compounds from EOs act as natural herbicides with several modes of action, including increasing lipid peroxidation^5,46^. The MDA accumulation remark here is crucial evidence supporting that CEO-based nanoemulsions have herbicidal potential on both weeds (*E. crus-galli* and *A. tricolor*) and may be utilized as natural herbicides.

#### Photosynthetic pigments

**Figure 10:**
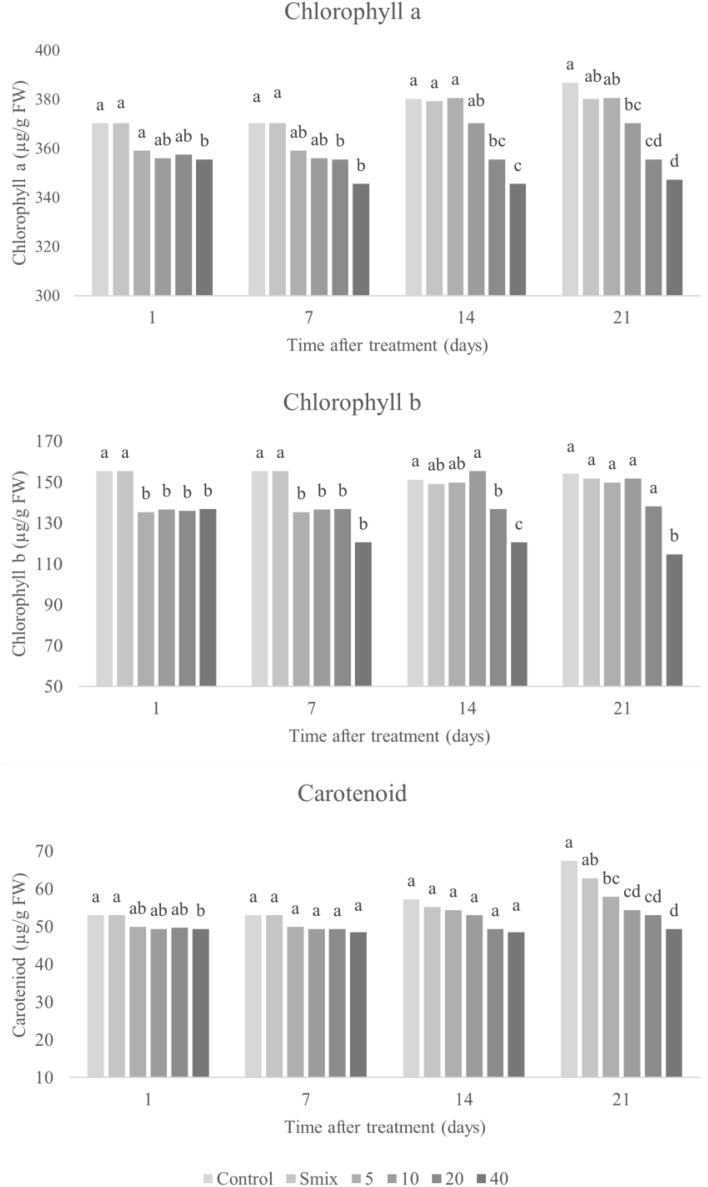
Chlorophyll a, b and carotenoid content in *E. crus-galli* leaves treated with surfactant mixture (Smix) (40 mL/L) and citronella essential oil nanoemulsion at different concentrations of citronella essential oil (5–40 mL/L) prepared using the microfluidizer at 20,000 psi for 7 cycles with surfactant mixture (Tween 60 and Span 60). Means with different letters within time after treatment are significantly different as indicated by Tukey’s test (*p* ≤ 0.05).

**Figure 11:**
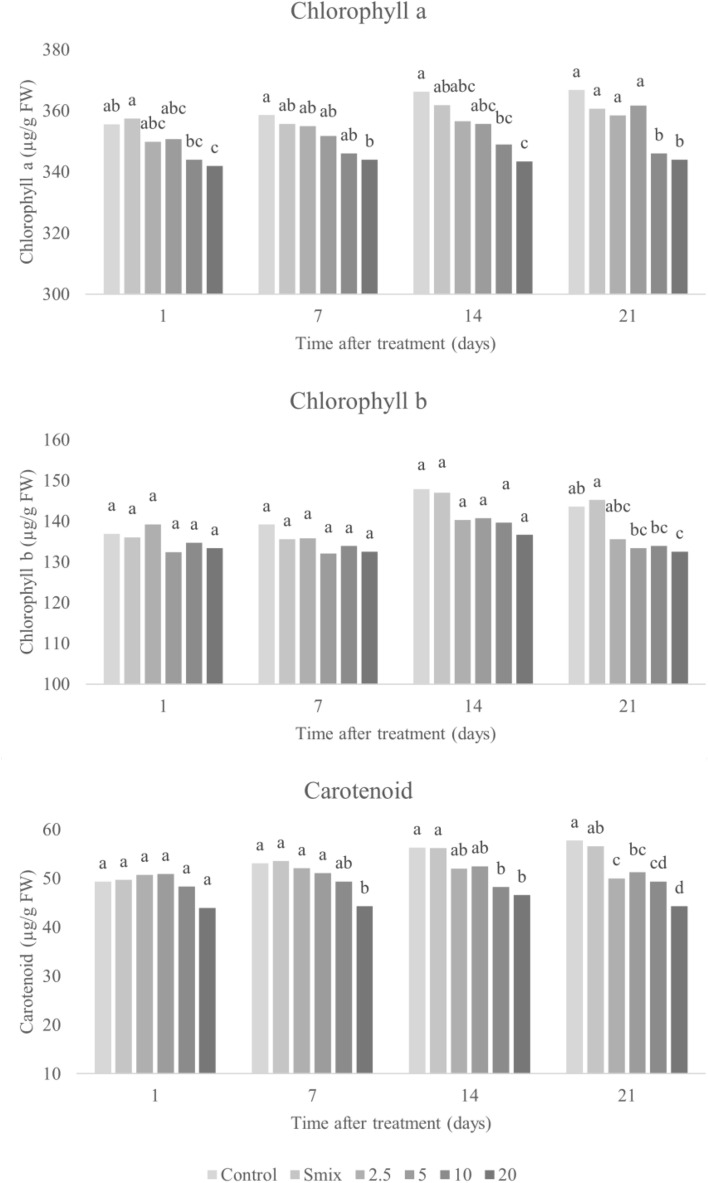
Chlorophyll a, b and carotenoid content in *A. tricolor* leaves treated with surfactant mixture (Smix) (20 mL/L) and citronella essential oil nanoemulsion at different concentrations of citronella essential oil (2.5–20 mL/L) prepared using the microfluidizer at 20,000 psi for 7 cycles with surfactant mixture (Tween 60 and Span 60). Means with different letters within time after treatment are significantly different as indicated by Tukey’s test (*p* ≤ 0.05).

Photosynthetic pigments are key to absorption of light energy to produce chemical energy during photosynthesis process, and hence are essential for electron transport process in photosystems I and II^53^. The effects of the nanoemulsion on photosynthesis pigment contents, were investigated at 1, 7, 14, and 21 days after foliar application to the tested plants. Treated leaves became pale yellow (data not shown), and photosynthetic pigment contents decreased in a dose-dependent manner (Figs. 10 and 11), with greater decrease as time passed. Significant differences from the control were observed in all pigments at EO concentrations of 5 mL/L and above.

Photosynthetic pigments react with ROS, which can be a possible explanation for pigment decrease in times of stress^5^ and aligns with our observations of increased electrolyte leakage and lipid peroxidation in treated leaves. The present results agreement with the findings of Ootani et al.^17^, who observed *C. nardus* and *Eucalyptus citriodora* EOs to reduce chlorophyll in treated weeds by up 50%. Other studies have likewise highlighted the phytotoxic effect of EOs on chlorophyll^2,14,43,46^. As chlorophyll is the critical pigment for capturing electrons in photosystem II^17^ and carotenoids protect against photooxidative damage^54^, pigment reduction may impede growth and allow escape of free radicals produced during photosynthesis processing, which can then cause damage.

According to the obtained results, evaluating the efficiency of the nanoemulsion of CEO demonstrated that the nanoemulsion had multiple modes of action that may correlate with their complex component mixture. In literature, CEO was used as a bioactive source for multiple applications of therapeutic uses, cosmetic, pharmaceutical industries antimicrobial, antifungal and enzyme inhibition^14,55^. Also, herbicidal activity is one of the bioactivities of CEO.

## Conclusion

In this research, a CEO based-nanoemulsion was fabricated by microfluidization method and characterized, then determined for its herbicidal efficacy against *E. crus-galli* and *A. tricolor* when applied as a foliar spray. The nanoemulsion affects both *E. crus-galli* and *A. tricolor* which represent narrow- and broadleaf weed plants, respectively. Therefore, this nanoemulsion may be a non-selective herbicide. This formulation has been shown to inhibit weed seed germination as a natural pre-emergence herbicide in a previous report. In this study, the formulation droplet presented the nanoscale size with narrow distribution. The smallest droplet size was obtained from the microfluidization condition at 20,000 psi and 7 cycles. The current report confirms that the CEO nanoemulsion may act as a post-emergence herbicide with multiple MOAs (decreased photosynthetic pigments and increased electrolyte leakage and MDA) in *E. crus-galli* and *A. tricolor* under the greenhouse pot test. Further, the efficiency of the nanoemulsion should be studied under field conditions as well. Moreover, CEO-based nanoemulsions should be tested for their toxicity compared to the use of chemical herbicides in the future.

## Data Availability

All data generated or analyzed during this study are included in this published article.
